# Phenotypic Consequence of Rearranging the N Gene of RABV HEP-Flury

**DOI:** 10.3390/v11050402

**Published:** 2019-04-29

**Authors:** Mingzhu Mei, Teng Long, Qiong Zhang, Jing Zhao, Qin Tian, Jiaojiao Peng, Jun Luo, He Jiang, Yingyi Lin, Zhixiong Lin, Xiaofeng Guo

**Affiliations:** 1 College of Veterinary Medicine, South China Agricultural University, Guangzhou 510642, China; meimz@126.com (M.M.); jerrylongt@126.com (T.L.); joanzhang@cm-agriculture.com (Q.Z.); zjwgq8912@163.com (J.Z.); tiansir89@163.com (Q.T.); m13560466761@163.com (J.P.); lj378483477@163.com (J.L.); 15212420597@163.com (H.J.); lamwingE@hotmail.com (Y.L.); 2 Guangdong Inspection and Quarantine Technology Center, Guangzhou 510623, China

**Keywords:** HEP-Flury, rabies virus, gene rearrangement, nucleoprotein

## Abstract

Nucleoprotein (N) is a key element in rabies virus (RABV) replication. To further investigate the effect of N on RABV, we manipulated an infectious cDNA clone of the RABV HEP-Flury to rearrange the N gene from its wild-type position of 1 (N-P-M-G-L) to 2 (P-N-M-G-L), 3 (P-M-N-G-L), or 4 (P-M-G-N-L), using an approach that left the viral nucleotide sequence unaltered. Subsequently, viable viruses were recovered from each of the rearranged cDNA and examined for their gene expression levels, growth kinetics in cell culture, pathogenicity in suckling mice and protection in mice. The results showed that gene rearrangement decreased N mRNA transcription and vRNA replication. As a result, all viruses with rearranged genomes showed worse replication than that of rHEP-Flury in NA cells at a MOI of 0.01, but equivalent or slightly better replication levels at a MOI of 3. Consequently, the lethality in suckling mice infected with N4 was clearly attenuated compared with rHEP-Flury. However, the protection to mice was not enhanced. This study not only gives us insight into the understanding of the phenotype of RABV N gene rearrangement, but also helps with rabies vaccine candidate construction.

## 1. Introduction

Gene rearrangement has been used to alter the genotypes of viruses that result in predictable changes in gene expression, which can be invaluable for gene function and control studies [1]. Previous work has demonstrated that rearranging the five viral genes of vesicular stomatitis virus (VSV), a prototype Vesiculovirus genus of the *Rhabdoviridae* family, could affect its relative protein expression levels and consequently alter phenotypes and lethality in mice infected with recombinant viruses [1,2]. Neither of the RNA type in rearranged viruses infected cells, nor the relative molar ratio of the proteins in mature virus particles were changed by gene rearrangement [3].

The Rabies virus (RABV), which is a member of the genus Lyssavirus of the family *Rhabdoviridae*, causes severe neurological disease and death in almost all kinds of mammals, including humans [4]. Its genome consists of a single negative-stranded RNA molecule of approximately 12 kb, which encodes five viral proteins in the order of 3′-N-P-M-G-L-5′ [5,6]. The RABV N protein, which is decisive for virus replication, tightly encapsidates the genomic and anti-genomic RNA of RABV to form the viral ribonucleoprotein (RNP) complex, known as the template for viral transcription and replication [6,7]. Moreover, purified N protein of fixed rabies virus can induce potent T helper cell responses resulting in long lasting and strong humoral immune responses against RABV [8,9]. Additionally, the amino acids at positions 273 and 394 in the N protein are important for both evasion of the host RIG-I-mediated antiviral response and pathogenicity [10,11,12].

In many aspects, transcription and replication of single-stranded, negative-sense RNA viruses are similar. Previous work has shown that the G mRNA of RABV ERA can be increased by 30% by switching the G gene position to the M gene position [13]. The RABV P protein can be reduced by translocating the P gene to the promoter distal position, which results in reduced lethality in mice [14,15]. We have also rearranged the P gene of RABV HEP-Flury and found that the P mRNA ratio was positively associated with its gene position, although the P mRNA was detected at almost the same amount as the N mRNA in HEP-infected BHK-21 cells [16]. Subsequently, we found that the viral lethality in mice was attenuated as the P mRNA ratio was downregulated [17].

In the present study, we first rearranged the N gene of HEP-Flury, by translocating it from its wild-type position of 1 to 2, 3, or 4, in the hope of reducing viral replication and therefore attenuating viral lethality. Then, we recovered viable viruses and characterized them to understand the relationship between gene expression and viral characteristics in detail, which is critical for the prevention and control of the disease.

## 2. Materials and Methods

### 2.1. Viruses, Plasmids, Cell lines and Animals

Two recombinant RABV strains were used in this study, rHEP-Flury (N1) and N2, of which the N gene was positioned at first site and second site respectively [17,18]. The plasmid pHEP-3.0 containing the full-length genome cDNA of HEP-Flury and the four helper plasmids, pH-N, pH-P, pH-G, and pH-L were a kind gift from Dr. Kinjiro Morimoto of the NIH in Japan [19]. For the in vitro assays, we used the Baby hamster kidney (BHK-21) cell line and Mouse neuroblastoma (NA) cell line as model, which were maintained in Dulbecco-modified essential medium (DMEM) (Gibco, Invitrogen, shanghai, China) with 10% fetal bovine serum (FBS) (Gibco, Shanghai, China) and RPMI1640 medium (Gibco, Invitrogen, Shanghai, China) with 10% FBS, respectively. For the in vivo assays, the specific pathogen free (SPF) female Kunming adult and pregnant mice were set as model, which were purchased from Center for Laboratory Animal Science, Southern Medical University of China (Guangzhou, China). The grouped mice were maintained under SPF conditions in Laboratory Animal Center of South China Agricultural University, and all the procedure involving animal ethics were conducted in compliance with NIH guidelines [20] and approved by the Institute of Animal Care and Use Committee of South China Agricultural University. All experiments requiring injection of RABVs were carried out in a special laboratory (biosafety level 2) designed for in vivo infectious experiments. Additionally, since none of the mice showed any clinical signs of rabies, they were all sacrificed by carbon dioxide inhalation after a certain survival time in accordance with the experimental schedule.

### 2.2. Construction of Full-Length cDNA Clones

We rearranged the N gene from proto-site (1st site) to 2nd, 3rd and 4th site with the aim to study the function of RABV N gene. An efficient homologous-recombinant-based ClonExpressTM MultiS one step cloning method was adopt to rearrange the N gene of RABV as previous described [17]. All RABV genes were rearranged from the beginning of the transcription start site (*AACA*) to the transcription end signal (the poly A signal *AAAAAAA*). First, we used inverse PCR to amplify the linearized vector for the cDNA construction. The linearized vector for plasmid N3 was amplified using the primers N3-VF (5′-*TATTAACATCCCTCAAAAGACTTAAGG*-3′; the 15–20 bp homologous sequences are underlined) and N3-VR (5′-*AGTTTTTTTCATATCGACTCCATGAC*-3′; the 15–20 bp homologous sequences are underlined). The linearized vector for plasmid N4 was amplified using the Primers N4-VF (5′-*TGTATACCAAAAGAACAACTAACAACAC*-3′; the 15–20 bp homologous sequences are underlined) and N4-VR (5′-*GCCTGTTTTTTTCACATCCAAGAG*-3′; the 15–20 bp homologous sequences are underlined). The primers for the amplification of genes are list in Table 1. Both the linearized vectors and target genes were amplified using Phusion High-Fidelity DNA Polymerase (Thermo Scientific, Waltham, MA, USA) in order to avoid mutation. Then, the amplified linear vector and target gene were ligated to construct the full-length cDNA using an efficient homologous-recombinant-based ClonExpress^TM^ MultiS one step cloning kit according to the manufacturer’s instructions (Vazyme Biotech, Nanjing, China). Finally, the plasmids were verified by PCR amplication and sequencing (Invitrogen, Guangzhou, China) in order to make sure the correction of the base sequence.

### 2.3. Rescue of the Rearranged Viruses from cDNA

The rearranged viruses were rescued, as previously described [19,21,22]. Briefly, the BHK-21 cells that pre-seeded in 12-well plates (Corning, NY, USA) were transfected with the plasmids containing the rearranged cDNA along with the four helper plasmids, pH-N, pH-P, pH-G, and pH-L using the SuperFect transfection reagent (Qiagen, Hilden, Germany) according to the manufacturer’s protocol. After a few weeks, the supernatants of transfected cells were collected and the existence of the rescued virus was examined via direct immunofluorescence assay (IFA) using FITC-conjugated antibodies against the RABV N protein (Fujirabio Diagnostics, Malvern, PA, USA). Subsequently, the viruses rescued successfully were passaged in NA cells. The gene orders of the rescued viruses were determined by reverse transcription (RT)-PCR after eight passages. Specific fragments were amplified using the primers D-F(*CTTAACAACAAAACCAAAGAAGAAGCA), P-R(CATCTCAAGATCGGCCAGACCG*), N-R(*TGAAGTTCGGTATAGTACTCC*), and M-R(*GTCCTCATCCCTACAGTTTTTC*) [17]. Further confirmation was done by sequencing.

### 2.4. Quantitative Real-Time PCR

To examine the transcription level of N, P, M, G, and L mRNAs, the viral genomic RNA (vRNA) level and leader RNA (LeRNA) level, the monolayers of NA cells were infected with rHEP-Flury and rescued viruses, respectively, at a multiplicity of infection (MOI) of 3 to make sure every cell was infected. The cells were washed with phosphate buffered saline (PBS) and harvested after 12 h incubation at 34 °C, then the total RNA was purified using a HiPure Universal RNA kit (Magen, Guangzhou, China) and reverse transcription (RT) was carried out using the Transcriptor First Strand cDNA Synthesis Kit (Roche, Basel, Switzerland) according to the manufacturer’s instructions. The transcription level of each gene was determined, as previously described [17]. Table S1 provides primer sequence details. A SYBR Green Master Mix (Vazyme Biotech CO., Ltd., Nanjing, China) was used for the real-time PCR (qPCR) and qPCR was carried out in a CFX384 Real-Time System (Bio-Rad, Hercules, CA, USA). The copy numbers of each target gene were normalized to the housekeeping gene beta actin (β-actin).

### 2.5. Western Blot

To investigate the expression level of the RABV N, P, M and G, Western Blot analysis was performed with monoclonal mouse anti-RABV N (Tongdian Biotech, Hangzhou, China), P, M and G (prepared by our lab) respectively. Briefly, the pre-seeded monolayers of NA cells were infected with rescued viruses respectively, and the cells were lysed to collect the total protein using RIPA buffer (containing 1× protease inhibitor cocktail, Beyotime, Shanghai, China) according to our previous work [23] after 12 h post-infection (hpi), the harvested protein was quantitated using a Pierce BCA protein assay kit (Thermo scientific, Waltham, MA, USA) and normalized into same concentration, 15 μL of proteins were separated by SDS-10% polyacrylamide gel electrophoresis (SDS-10% PAGE) followed by western blotting analysis, the relative expression level was normalized with β-actin (Beyotime Biotech, Shanghai, China) expression. Chemiluminescence analysis using BeyoECL plus (Beyotime Biotech, Shanghai, China) was performed, as instructed by the vendor and the protein bands were evaluated by Image-Pro plus.

### 2.6. Virus Growth and Spread Assays

To investigate the virus growth, confluent NA cell monolayers were infected with one of the rescued viruses at a MOI of 0.01 for multi-step growth curves or 3 for single-step growth curves. The infected cells were maintained at 34 °C with fresh RPMI 1640 containing 5% FBS, and the culture medium were harvested at the indicated time points, the virus was titrated in triplicate on NA cells via direct fluorescent antibody assays as described previously [24]. For the viral spread, the NA cells were infected with rescued viruses at a MOI of 0.005 for 72 h incubation in total at 34 °C, then the infected cells were observed under a fluorescent microscope with the FITC anti-rabies monoclonal globulin (Fujirebio, Malvern, PA, USA) every 12 h [25]. 

### 2.7. Cell Apoptosis by Flow Cytometry

To investigate the cell apoptosis induced by the RABVs, the pre-seeded NA cells were infected with rescued RABVs at a MOI of 3, the cells were harvested and stained with annexin-V and propidium iodide (PI) using annexin V-FITC/PI kit (Bestbio, Shanghai, China) at 24 hpi following the manufacturer’s instruction. The cell apoptosis was evaluated with a Beckman FC 500 flow cytometry (Beckman Coulter, Brea, CA, USA), and data were analyzed with CXP software. 

### 2.8. Lethality in Suckling Mice

Suckling mice aged 1 to 3 days old served as a sensitive model for comparing the relative lethality of rHEP-Flury and its mutants, as rHEP-Flury is fatal for suckling mice and nonpathogenic to the adult mice after IC inoculation [26]. Firstly, all the viruses were titrated to 10^5.5^ FFU/mL with RPMI 1640 medium, then grouped of 12 mice were inoculated with 20 μL of serial 10-fold dilution of the virus via intracerebral route (IC), or RPMI 1640 for mock infection using a 50 μL glass microsyringe, observed daily, the mice that died within the beginning 4 days due to the shock response were ignored. The LD_50_ for each virus was calculated with the Reed and Muench method.

### 2.9. Immunogenicity of the Rescued RABVs

To determine the immunogenicity of the RABVs, grouped of 10 adult Kunming mice (6–8 weeks) were immunized once via intramuscular (IM) route at quadriceps femoris with 50 μL of 10^5^, 10^4^ or 10^3^ FFU rescued RABVs using the 1 mL syringe. Peripheral blood was harvested from orbital sinus at 21 days post-immunization (dpi) and the purified serum sample were heated at 56 °C for 30 min to inactivate the complement. The antibody levels were evaluated using a Serelisa® Rabies Ab Mono Indirect kit (Synbiotics, Lyon, France) following the manufacture’s introductions, and the mice were considered as protected while the antibody level was >0.6 EU/mL. Then, the immunized mice were challenged by IC with 30 μL of 50 LD_50_ of the challenge virus standard (CVS)-24 and observed for successive 28 days, the mice that died within the first 4 days were neglected. To investigate the peripheral antibody duration in adult mice, grouped of 5 mice (6–8 weeks) were inoculated IM with diluent or 10^5^ FFU individual viruses. After virus inoculation, the blood was collected at weekly intervals to determine antibody levels, as described before.

## 3. Results

### 3.1. Structure of the Rearranged RABV

**Figure 1:**
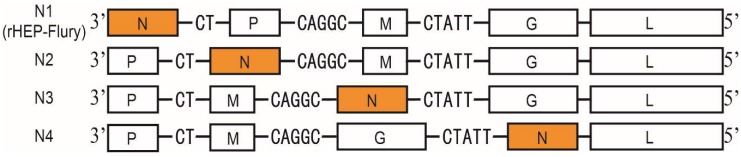
Genomic structure. The genomic structure of the rescued RABVs and the rearranged gene was yellow highlighted.

To study the relationship between gene expression and viral characteristics, the function of N gene of HEP-Flury was studied by rearranging the N gene positions from proto-site (1st site) to 2nd, 3rd, and 4th site, respectively, with other aspects of the viral nucleotide sequences remaining unaltered. The recovered viruses were designated as N1 (rHEP-Flury), N2, N3 and N4 according to the specific position that the N gene was at, and the genomic structures of the rearranged RABVs were shown in Figure 1. To validate the gene order of the rearranged RABVs, RT-PCR was conducted and the observed sizes of the amplified fragments were exactly as predicted (Figure S1), the accuracy of the amplicons was proved by Sanger sequencing. The results above indicated that the gene order of the recovered viruses was in accordance to the cDNA clones from which they were rescued.

### 3.2. Effect of Gene Rearrangement on mRNA and Protein Expression

The synthesis of viral RNAs and proteins in infected NA cells at 12 h post infection was examined to ascertain whether N gene rearrangement affected gene expression, as we expected. At the same time, we stained the NA cells with FITC anti-rabies monoclonal globulin and confirmed the infection of every cell.

**Figure 2:**
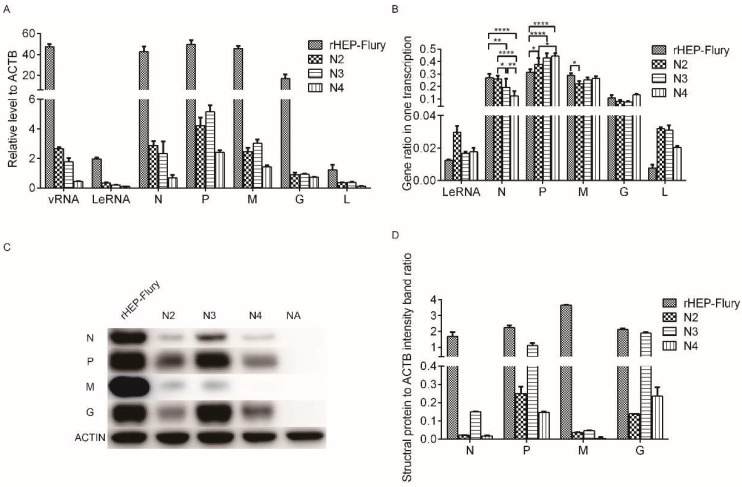
Transcription and translation level of the RABVs gene. (**A**) Transcription level of structural gene of rescued RABVs determined by RT-qPCR. The relative amount of individual viral RNAs was normalized by housekeeping gene, β-actin. The data display as mean ± SD, *n* = 3. (**B**) The ratios of LeRNA, N mRNA, P mRNA, M mRNA, G mRNA, or L mRNA in transcription. The individual RNA ratios were calculated relative to all structural genes plus LeRNA: LeRNA + N + P + M + G + L. Data are mean ± SD, *n* = 3. The statistical analyses were performed using 2way ANOVA, significant differences between groups were denoted by * *p* < 0.05; ** *p* < 0.01; *** *p* < 0.001; **** *p* < 0.0001. (**C**) The expression of viral protein at 12 hpi was determined with Western blotting using monoclonal antibodies against RABV N, P, M, and G, respectively, and monoclonal antibody against β-actin as reference gene. (**D**) The densitometry of the Western blotting (**C**) was analyzed with the Image-Pro Plus 6.0 software and the expression of N, P, M, or G protein was normalized with β-actin.

N mRNA in the infected cells decreased substantially compared with the rHEP-Flury level as the N gene was moved successively away from the promoter in the N2, N3, and N4 viruses, as we expected. Additionally, vRNA synthesis was reduced as similar as the N mRNA (Figure 2A). This was consistent with N protein synthesis, which has been deemed control the RNA replication levels [27,28]. The P, M, G, and L mRNA transcription levels were reduced as the vRNA was reduced, presumably as a secondary effect because of the decrease in replication. For the N3 virus, its P, M, G, and L mRNA levels were equal or more than that of the N2 virus. This might have been due to the increase in the M mRNA ratio, which stimulates viral replication [29]. Further correlation analysis between the mRNAs and vRNA or LeRNA also showed that they had significant correlations (correlation coefficient >0.9, *P* < 0.01). This revealed that the transcription of the RABV mRNAs were mainly affected by viral replication, as RABV only encodes five subgenomic mRNAs, which are translated into five proteins, all of which are components of the mature virions [30]. 

To further confirm the effects of gene position on mRNA transcription without the influence of viral replication, we analyzed the mRNA ratios for each virus, i.e., the ratio of each transcript was calculated relative to all mRNAs plus LeRNA, which included, LeRNA + N mRNA + P mRNA + M mRNA + G mRNA + L mRNA in every virus. The data showed that the ratio of N mRNA decreased substantially compared with the rHEP-Flury level as its gene was moved successively away from the promoter in viruses N2, N3, and N4, although previous work has shown that the N and P mRNA of rHEP-Flury were quantitatively similar in infected BHK cells [16]. Consistent with this decrease, an increase in the P mRNA ratio was observed with viruses N2, N3 or N4, in which the P gene was moved one position closer to the promoter. Additionally, an increase in the G mRNA ratio was observed with virus N4, in which the G gene was moved one position closer to the promoter. Notably, the LeRNA and L mRNA ratios were upregulated and varied in N gene rearranged virus infected NA cells, even though their positions were not changed (Figure 2B). Therefore, we presumed that the N, P, M, and G gene positions mainly affected the gene transcription ratio in one complete transcription.

The amounts of viral proteins were qualitatively similar with their mRNAs as the translation efficiency was mainly regulated by the level of transcription (Figure 2A,D). However, the N and G translation efficiency of N2 were lower than others. There might be some other factors in the N2 virus that inhibit translation; therefore, further work is needed.

### 3.3. Replication and Spread of Viruses with Rearranged Genomes

**Figure 3:**
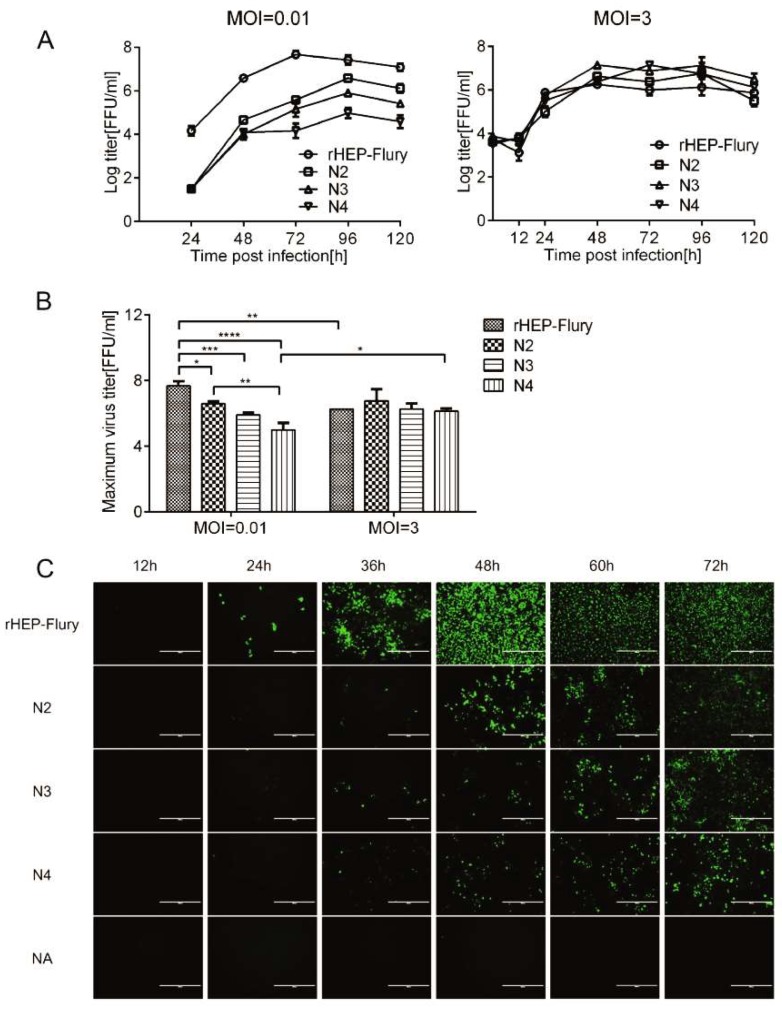
The growth kinetic curves and spread of the RABVs in NA cells. (**A**) Multi-step and one-step growth curves of the rescued RABVs are displayed. The virus titres were determined at the indicated intervals were assayed in triplicate, and the results were averaged. (**B**) The maximum titer of the rescued RABVs in NA cells at MOIs of 0.01 or 3. Statistical significance was analyzed by two-way ANOVA and the asterisks denote significant differences (* *p* < 0.05; ** *p* < 0.01; *** *p* < 0.001; **** *p* < 0.0001). (**C**) The viral spread in NA cells. The infected cells were stained with the FITC anti-rabies monoclonal globulin at indicated time points and examined under fluorescence microscope, for each group, the presentative image out of three replicates was shown.

Transcription was reduced after rearranging the N gene, as described above. To investigate whether decreased transcription affects viral growth and spread, NA cells were infected with rHEP-Flury and rearranged viruses. Analysis of progeny virus production in cell culture showed that the viral replication declined as the N gene was translocated downstream of its normal promoter proximal position at a MOI of 0.01, as predicted (Figure 3A). At a MOI of 3, we found that the viral titer at 24 hpi was consistent with the M mRNA ratio in one complete transcription. Therefore, we assumed that the M mRNA ratio that increased in one complete transcription might promote the viral replication in a single cell. The maximum titer showed that the recombinant viruses could compensate for the defects of the populations with rearranged genomes at high MOI, but not at low MOI, which was demonstrated in VSV (Figure 3B) [31,32]. Here, we guessed that the M gene might regulate viral replication by regulating gene translation efficiency.

A viral spread assay in NA cells showed that viral spread varied as the viruses grew at a MOI of 0.01 (Figure 3C). Suppressed viral replication of rescued viruses likely reduced viral growth rate and led to the subsequent reduction in NA cells spread. At 36 hpi, the number of N4-infect cells was more than that of N2 or N3-infected cells. Additionally, its progeny virus titer was as high as N3 at 48 hpi, which might be due to increased G mRNA, which promotes virus uptake and limits the viral spread [33]. 

### 3.4. Cell Apoptosis

**Figure 4:**
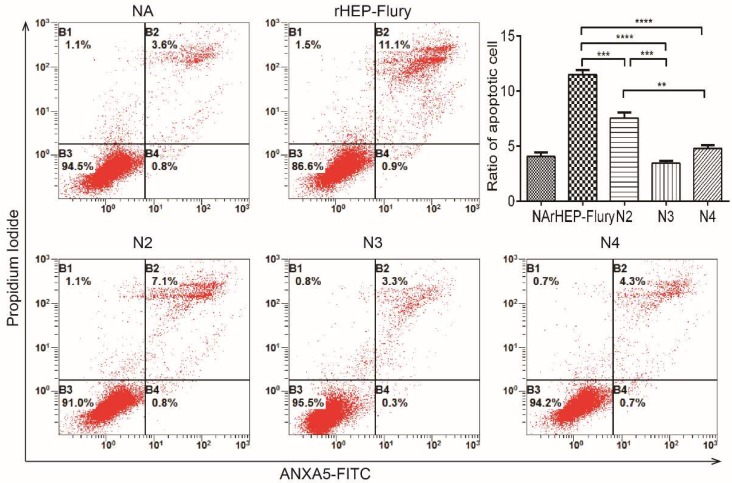
Flow cytometry to detect the cellular apoptosis. NA cells were infected with rHEP-Flury and the recovered viruses at a MOI of 3. Cell apoptosis was quantified using an Annexin V-FITC apoptosis kit. Cells in early apoptosis and dead cells were shown as the percentage of ANXA5-FITC and Ptdlns cells of the total cells. Mean ± SD of three independent experiments are shown. The statistical significance was denoted by ** *p* < 0.01; *** *p* < 0.001; **** *p* < 0.0001.

To investigate the function of RABV N gene exerted on cellular apoptosis, the infected NA cells were determined with annexin-V and PI. We have observed that the percentage of early stage apoptotic cells infected with rHEP-Flury was around 12.0% at a MOI of 3 plus late stage apoptotic or even necrotic cells, which was significantly greater than that induced by the N gene rearranged viruses (Figure 4). Briefly, the ability to induce cellular apoptosis of variant RABVs was negatively related to the distance between N gene to the genomic promotor, in which rHEP-Flury was greater than N2, N2 was greater than N3. Interestingly, N4 performs no significant difference with N3, it should be owing to that G gene which was a major factor for cytotoxicity was one position closer to the promoter [34]. Anyway, above results suggest that the RABV N gene was involving in cellular apoptosis in terms of viral replication.

### 3.5. Lethality in Suckling Mice

**Figure 5:**
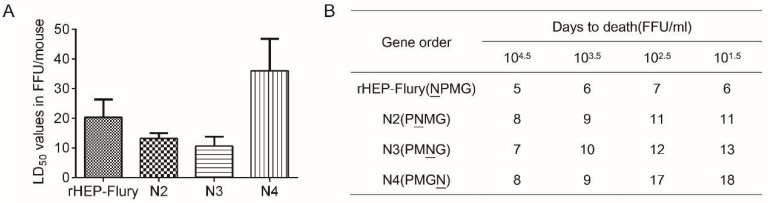
Lethality of RABVs in suckling mice. (**A**) The bar chart of the LD_50_, which was determined from mortality by groups of 12 mice inoculated IC with five serial, 10-fold dilutions of the viruses. (**B**) Onset of death after intracerebral inoculation of four serial dilutions.

According to previous work, translocating the N gene of VSV from its normal position to the second, third or fourth positions could reduce N gene expression, thus successfully attenuating viral lethality [1]. In this study, we also investigated whether N rearrangement in RABV affected pathogenicity. By IC inoculation, the LD_50_ dose of the rescued viruses, N2 and N3, required a slightly lower (0.5-fold) dose than rHEP-Flury. However, the N4 virus required a higher (1.4-fold) dose (Figure 5A). This demonstrated that the lethality in suckling mice following N2 and N3 infection was enhanced, although their replication ability was reduced.

From Figure 5B, the time to mortality onset at doses of 10^4.5^ FFU/mL to 10^1.5^ FFU/mL per mouse showed that rHEP-Flury-inoculated suckling mice started dying at day 5 post inoculation. When the N gene translocated from its normal position to the second, third, or fourth positions, the onset of death gradually occurred later. Additionally, the time to death was delayed more clearly as the dose was decreased. This was consistent with viral spread in the NA cells. We speculated that the ability of viral spread mainly affected the time to death.

### 3.6. The Ability of Rearranged Viruses to Protect against Wild-Type Challenge

**Figure 6:**
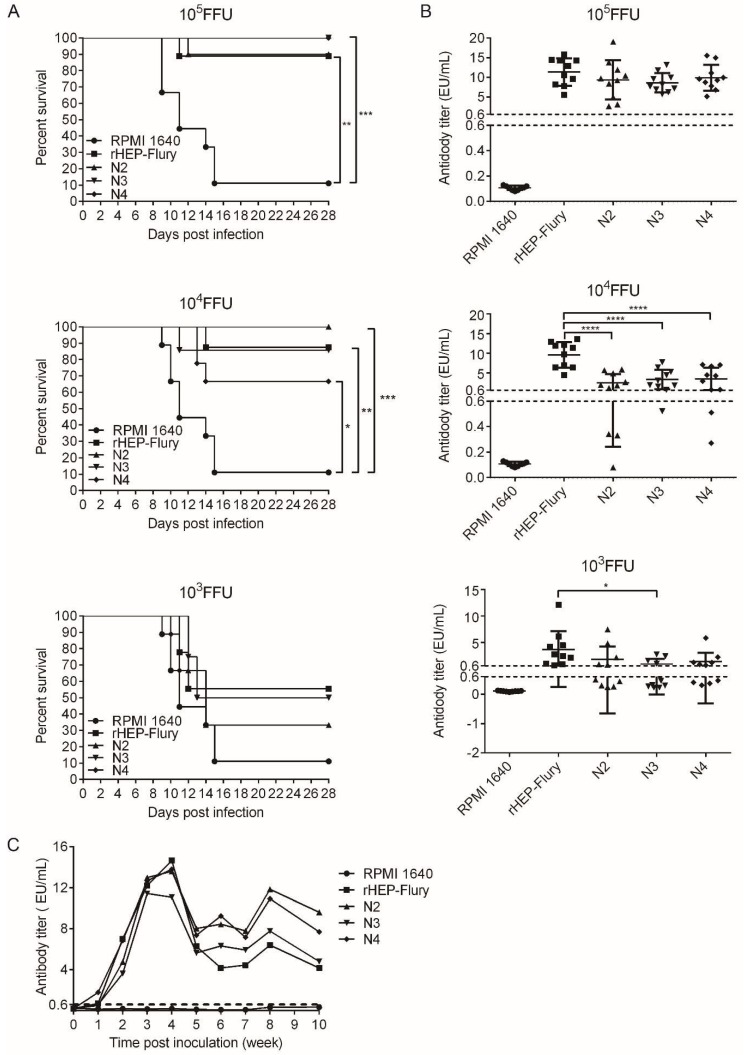
The antibody titres and the ability to protect against lethal challenge. (**A**) Survival of mice after immunized with rescued RABVs or culture medium for mock *via* an IM route. The statistical significance of survival rates was analyzed for with Kaplan-Meier plots (*n* = 10 in each group; by log-rank test). (**B**) The titre of anti-RABV antibody in serum at 21 dpi prior to challenge. The serum antibody titre of N2, N3, or N4 was compared against rHEP-Flury using one-way ANOVA. Statitically significant differences were denoted using asterisks (* *p* < 0.05; ** *p* < 0.01; *** *p* < 0.001; **** *p* < 0.0001) between the different groups. (**C**) Antibody kinetics of viruses with N gene translocations. The serum samples of inoculated mice were harvested to analyze the antibody titer.

To test whether N gene translocation affected the ability to elicit a protective immune response, mice were immunized by IM inoculation with 10^5^, 10^4^, or 10^3^ FFU of the rHEP-Flury or the rescued viruses, N2, N3, and N4. The surviving animals were challenged 21 days later by IC inoculation with 50LD_50_ of CVS-24. The protection mediated by all the viruses was significantly higher than the control at the 10^4^ and 10^5^ FFU doses. However, the protection decreased as the dose was reduced, as there was no significant difference between the viruses and control at the 10^3^ FFU dose. Notably, the protection mediated by RABV N4 was decreased, which might be because of its poor replication ability (Figure 6A). 

Serum antibody measurements prior to challenge on day 21 showed that the antibody level present in the serum of the immunized animals correlated with the level of apoptosis at the 10^5^ FFU dose. This might indicate that the speed or extent of apoptosis was directly determined by the magnitude of the antibody response [35]. At the 10^4^ FFU dose, the antibody level present in the serum of the rHEP-Flury immunized animals were significantly higher than the others, which might be because the rHEP-Flury with the wild-type gene order was the most fit for growth (Figure 6B) [36].

### 3.7. The Antibody Level Duration in Mice

The serum antibody levels showed that the antibody kinetics of all the viruses were similar. They reached a peak at week 4, then decreased and were maintained at a level that was more than 0.6 EU/mL for 10 weeks post inoculation (Figure 6C), and from the curve, we assume that the viral neutralization process begins between weeks 3 and 4 and mainly happens between weeks 4 and 5, which caused the antibody reduction in the serum. The antibody levels against the N gene rearranged viruses were higher than those against rHEP-Flury five weeks later.

## 4. Discussion

The results presented above show that the RABV N gene can be rearranged from its promoter-proximal position of 1 to 2, 3, or 4 in the genome and infectious viruses can be successfully recovered. It was proven it was not necessary for N gene fixed on position 1, the transmission of N gene to other genomic site facilitates RABV replication as well. Subsequently, the N mRNA transcription decreased as predicted by the progressive transcriptional attenuation model. The vRNA synthesis in cells infected with N rearranged viruses decreased, along with their mRNA levels. This was consistent with previous work on the N protein, which is the most important element for viral replication and can control the RNA replication levels [5,37]. 

Meanwhile, we found that the LeRNA and L mRNA ratios increased in the N gene rearranged viruses. LeRNA can bind the host protein La which is normally found to be associated with RNA polymerase Ⅲ precursor transcriptions, that may inhibit cellular RNA synthesis [38], thus inhibiting viral replication. Recently, studies also showed that the pathogenic RABV LeRNA can interfere with the binding of RABV nucleoprotein with genomic RNA, thus inhibiting the RABV replication [39]. The L protein of the attenuated vaccine strain, SAD B 19, can bind to dynein light chain 1 (DLC1), which acts as a transcription enhancer [40]. Moreover, attenuated expression of L was approved to result in the downregulation of virus replication and transcription, which appears to be the self-limiting strategy of RABV HEP 5.0 to optimize gene expression and supported prolonged host cell survival [41]. Here, the increased ratio of LeRNA and L mRNA might suppress the viral propagation via these mechanisms. Interestingly, we found that the P mRNA ratio increased with the downregulation of N mRNA ratio, which was found in P gene rearranged viruses as well [17]. We speculate that there are some interactions between the N and P proteins that influence their ratios; therefore, further study is needed. 

Subsequently, the infectious virus production in cell culture was reduced in increments up to four orders of magnitude with virus N4 at a MOI of 0.01. While at a MOI of 3, the maximum rHEP-Flury titer was lower than that at a MOI of 0.01, which might be due to the strong apoptosis effect, although rHEP-Flury does not cause toxicity in NA cells at a MOI of 0.01 [22,23]. For N4, in which the G gene was translocated one position toward the promoter, its G mRNA ratio increased. Consequently, increased glycoprotein expression lead to more efficient receptor-mediated uptake in the early stages of infection [22,42]. Additionally, the G protein of attenuated rabies virus could induce apoptosis in the central nervous system, consequently limiting its viral spread [43,44,45]. This may have caused the higher NA cell infection rate at 36 h post infection and lower maximum titer.

Although the viral replication ability was successfully repressed, previous work also showed that the pathogenicity of RABV correlated to an increase in RABV mRNA levels and live viral load in the brain, as well as to an accelerated spread to brain regions [46], and only the pathogenicity of N4 was reduced. When we rearranged the N gene, the P gene was passively translocated one position closer to the promoter. The RABV P protein can interrupt IFN transcription, which consequently increases viral pathogenicity [47,48]. Previously, we also demonstrated that the P mRNA ratio positively associated with viral pathogenicity [17]. Moreover, the decrease in apoptotic cells also contributed to the increase in pathogenicity [49,50,51]. All of these changes might enhance the pathogenicity of N2 and N3. For N4, the G mRNA ratio and ability to induce apoptosis were increased; however, its spread in NA cells was interrupted. Additionally, apoptosis induction has been indicated to be an innate mechanism in which the host restricts viral spread and consequently attenuates RABV pathogenicity [52]. This might be the main factor that attenuates pathogenicity, although its P mRNA ratio was the highest in single virus-infected cells. For N2 and N3, they could infect all over the cells, although their cell-to-cell spread was slower. Additionally, we infected the suckling mice via an IC route, which may have prevented the host immune response. 

The protection mediated by the viruses decreased as the doses were reduced, which might because we inoculated with living viruses, in which immune responses correlated with viral replication in host cells. Additionally, the protection mediated by N4 might have been decreased because of its poor replication ability. At a dose of 10^5^ FFU, when the influence of viral replication could be neglected, the protection mediated by the N gene rearranged viruses was a little better than rHEP-Flury. This might because of the increase in LeRNA ratio, which could activate more dendritic cells [53]. The antibody level duration experiments in mice also showed that N gene rearranged RABVs induced higher antibody levels than rHEP-Flury, 5-weeks postimmunization, which might be effective antibodies that can help clear RABV CVS-24.

In summary, the replication and spread abilities of RABV rHEP-Flury can be successfully reduced via N gene rearrangement. Additionally, the lethality in suckling mice following variant virus N4 infection was clearly reduced because of the interruption in viral spread. Subsequently, we might be able to improve the maximum titer by increasing the N protein expression in vitro, which might provide a better vaccine candidate because of its irreversible rearrangement.

## Figures and Tables

**Table 1:** Oligonucleotides used for construction of rabies virus genome cDNAs ^a^.

Primer Name	Sequence (5′–3′)	Use
3-M-F	5′-GATATGAAAAAAACTAACACCACTAATAAAATGAAC	Construction for N3 plasmid
3-M-R	5′-GTAGGGGTGTTGCCTGTTTTTTTCACATCCAAGAGG	
3-N-F	5′-AGGCAACACCCCTACAATGGATGCCGACAAG	
3-N-R	5′-TGAGGGATGTTAATAGTTTTTTTCATGATGGATATAC	
4-G-F	5′-GTGAAAAAAACAGGCAACATCCCTCAAAAGACTTAAGGA	Construction for N4 plasmid
4-G-R	5′-GTAGGGGTGTTAATAGTTTTTTTCTCGACTGAAATGC	
4-N-F	5′-TATTAACACCCCTACAATGGATGCCGAC	
4-N-R	5′-GTTCTTTTGGTATACAGTTTTTTTCATGATGGATATAC	

^a^ 15–20 bp homologous sequences are underlined.

## Supplementary Materials

The following are available online at https://www.mdpi.com/1999-4915/11/5/402/s1, Figure S1: The nucleotide gel graph that showed the specificity of gene of the rescued RABVs. Lane M: Marker DNA fragments with sizes as indicated. Lanes 1, 4, 7, 10, and 13: PCR products with primers D-F and P-R.; Lanes 2, 5, 8, 11, and 14: PCR products with primers D-F and N-R.; Lanes 3, 6, 9, 12, and 15: PCR products with primers D-F and M-R; Table S1: Primers used for qRT-PCR and cDNA synthesis.
