# Supplementary material for: Phenotypic Consequence of Rearranging the N Gene of RABV HEP-Flury
Source: Viruses. 2019 Apr 29;11(5):402. doi: 10.3390/v11050402 (PMC6563252; doi:10.3390/v11050402)
Supplement: Supplementary file 1 [file viruses-11-00402-s001.pdf]

Supporting information

S1 Table. Primers used for qRT-PCR and cDNA synthesis.

Gene order	D-F and P-R(bp)	D-F and N-R(bp)	D-F and M-R(bp)
rHEP-Flury	1570	677	2537
N2	144	1670	2537
N3	144	2480	1108
N4	144	4241	1108

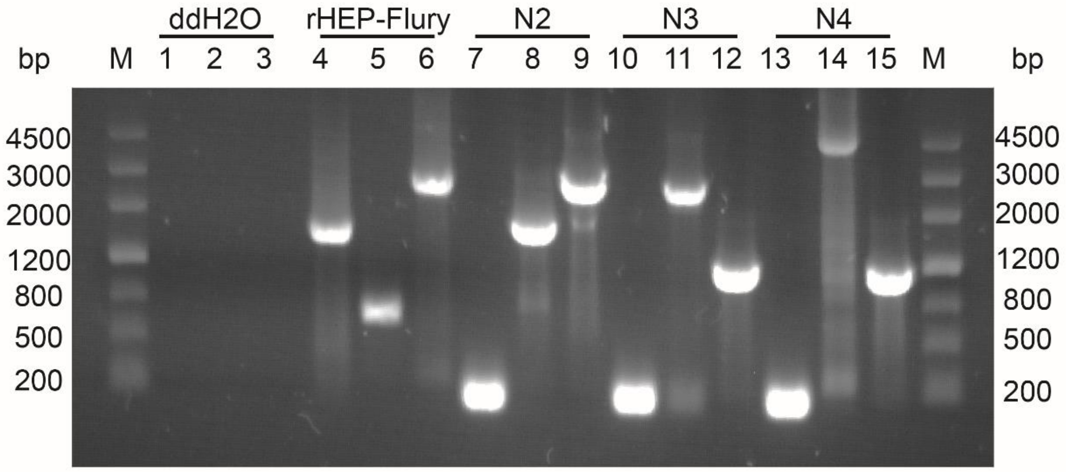

S1 Figure. The nucleotide gel graph that showed the specificity of gene of the rescued RABVs. Lane M: Marker DNA fragments with sizes as indicated. Lanes 1, 4, 7, 10, and 13: PCR products with primers D-F and P-R.; Lanes 2, 5, 8, 11, and 14: PCR products with primers D-F and N-R.; Lanes 3, 6, 9, 12, and 15: PCR products with primers D-F and M-R.
